# The Role of Bacterial Extracellular Vesicles in the Immune Response to Pathogens, and Therapeutic Opportunities

**DOI:** 10.3390/ijms25116210

**Published:** 2024-06-05

**Authors:** Eliud S. Peregrino, Jessica Castañeda-Casimiro, Luis Vázquez-Flores, Sergio Estrada-Parra, Carlos Wong-Baeza, Jeanet Serafín-López, Isabel Wong-Baeza

**Affiliations:** 1 Posgrado en Inmunología, Escuela Nacional de Ciencias Biológicas (ENCB), Instituto Politécnico Nacional (IPN), Mexico City 11340, Mexico; eliudsp.eb@gmail.com (E.S.P.); jcastanc@gmail.com (J.C.-C.); 2 Departamento de Inmunología, Escuela Nacional de Ciencias Biológicas (ENCB), Instituto Politécnico Nacional (IPN), Mexico City 11340, Mexico; sestradap07@hotmail.com (S.E.-P.); jeaserafin@hotmail.com (J.S.-L.); 3 Departamento de Bioquímica, Escuela Nacional de Ciencias Biológicas (ENCB), Instituto Politécnico Nacional (IPN), Mexico City 11340, Mexico; valu_868@hotmail.com (L.V.-F.); charlywong@icloud.com (C.W.-B.)

**Keywords:** extracellular vesicles, outer membrane vesicles, bacterial infection, vesicle-based vaccines

## Abstract

Pathogenic bacteria have several mechanisms to evade the host’s immune response and achieve an efficient infection. Bacterial extracellular vesicles (EVs) are a relevant cellular communication mechanism, since they can interact with other bacterial cells and with host cells. In this review, we focus on the EVs produced by some World Health Organization (WHO) priority Gram-negative and Gram-positive pathogenic bacteria; by spore-producing bacteria; by *Mycobacterium tuberculosis* (a bacteria with a complex cell wall); and by *Treponema pallidum* (a bacteria without lipopolysaccharide). We describe the classification and the general properties of bacterial EVs, their role during bacterial infections and their effects on the host immune response. Bacterial EVs contain pathogen-associated molecular patterns that activate innate immune receptors, which leads to cytokine production and inflammation, but they also contain antigens that induce the activation of B and T cell responses. Understanding the many effects of bacterial EVs on the host’s immune response can yield new insights on the pathogenesis of clinically important infections, but it can also lead to the development of EV-based diagnostic and therapeutic strategies. In addition, since EVs are efficient activators of both the innate and the adaptive immune responses, they constitute a promising platform for vaccine development.

## 1. Introduction

Numerous discoveries have been made by chance. Such was the case with the discovery of extracellular vesicles (EVs), which were described for the first time by Chargaff, who analyzed the procoagulant effect of the fraction sedimented at 31,000 g of platelet-free plasma [1]. Later, Chargaff and West identified that prothrombotic proteins and *tiny blood corpuscles* related to this procoagulant activity could exist in this plasma fraction [2]. Finally, Wolf used electron microscopy to observe a *platelet powder* taken from ultracentrifuged plasma, distinguishing this *powder* as an element of smaller size than platelet granules; this was the first observation of EVs [3].

One of the first functional descriptions of EVs was made by Johnstone, who concluded that EVs were a means for the elimination of waste proteins by identifying transferrin receptor-loaded vesicles in reticulocytes [4,5]. Since then, numerous groups have identified vesiculation in mammalian cells, algae, yeast, free-living bacteria, and commensals [5], suggesting, in addition, novel functions of EVs.

In the context of infectious disease immunology, Kay et al. did some of the first work demonstrating that EVs derived from *Porphyromonas gingivalis* inhibited chemotaxis and had a cytotoxic effect on human neutrophils [6]. In contrast, while analyzing the possible involvement of EVs as an immunological subcomponent, Raposo used a B cell line, which he stimulated with a peptide from *Mycobacterium leprae*, identifying that the stimulated B cell line expressed EVs loaded with bacterial peptides and that they were able to activate antigen-specific T cells [7].

Current evidence shows that, during pathologies, altered cells, whether due to metabolic, carcinogenic, or infectious processes, produce EVs that modulate the host response; moreover, the molecules contained in EVs and the vesicles themselves have been analyzed as a strategy for the development of vaccines or drug carriers [5].

The evolution of research on EVs and their role in the pathology and homeostasis of individuals and pathogenic microorganisms has had a turning point since these subcellular elements were conceived solely as a means of eliminating waste products and began to be analyzed as a primary mechanism for cellular communication [5,8]. In this review, we will analyze the most recent evidence on EVs as an important part of the immunology of infectious diseases of bacterial origin. Many studies and reviews in this area focus on the EVs produced by infected host cells. Here we will focus on the EVs produced by the pathogenic bacteria, and summarize the current knowledge about the role of these EVs during bacterial infections, their immunological effects, and their possible use as treatments and vaccines.

## 2. Extracellular Vesicles

The first studies on EV production date back to the 1940s, with experiments on the analysis of EVs in reticulocytes by Chargaff et al. [1,2]. Since then, it has been shown that the vesiculation process is not only present in human cells but also in other eukaryotes such as plants, algae, and fungi, and in organisms of the archaeal kingdom and bacteria [9]. The growth of the study of EVs led to the founding of the International Society for Extracellular Vesicles (ISEV) in 2011 and the establishment of the minimal information guidelines for studies of extracellular vesicles (MISEV) in 2014 [9], which was last updated in 2023 [9,10]. These guidelines have allowed researchers to deepen the concept of EVs as structures with cell membranes or cell wall components or both, transporting molecules exported to the extracellular medium [5].

**Figure 1:**
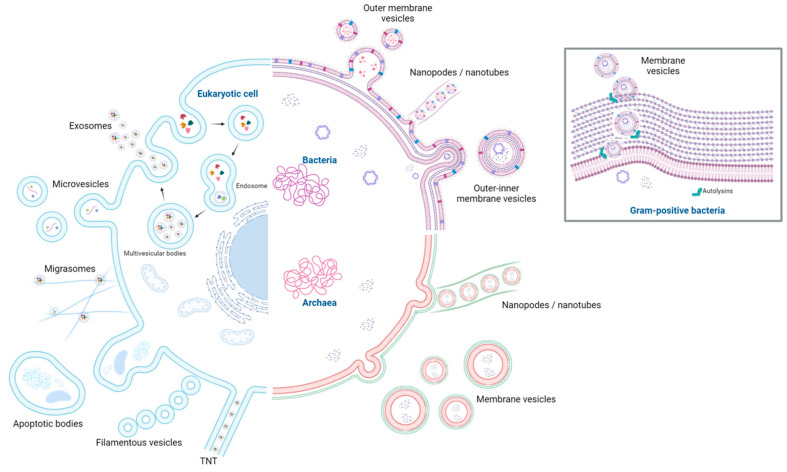
**Vesiculation in Eukarya, Archaea, and Bacteria.** Eukaryotic organisms produce exosomes, microvesicles, and apoptotic bodies; exosomes are generated from multivesicular bodies; microvesicles are generated by membrane pinching; and apoptotic bodies are produced during cell death. EVs have also been described in filaments (migrasomes), concatenated (apoptopodia), and transferred in cytoplasm bridges (tunnelling nanotubes, TNT). In bacteria, vesiculation occurs by lytic and non-lytic routes, where vesicles derived from the inner or outer membrane or both are distinguished. In addition, the production of EVs in nanopodia has been proposed; the inset of the figure shows the proposed process for vesiculation of Gram-positive bacteria. In Archaea, vesiculation occurs by membrane pinching and by nanopodia. References: Guerrero-Mandujano et al. [12]; Dean et al. [13]; and Palacios et al. [14]. Created with BioRender.com.

The biogenesis of EVs has been constantly studied because it is present in all three domains of life (Figure 1), but much EV research has focused on eukaryotic EVs. It is speculated that vesiculation is a process that has been present since the common evolutionary ancestor of all living beings [9,11].

In the Archaea domain, both Crenarchaeota (C-AEV) and Euryarchaeota (E-AEV) produce EVs. In the Bacteria domain, three types of EVs are produced by Gram-negative bacteria: outer membrane vesicles (OMV), outer-inner membrane vesicles (O-IMV) and explosive outer membrane vesicles (E-OMV). Cytoplasmic membrane vesicles (CMV) are produced by Gram-positive bacteria. The traditional EVs of eukaryotes are exosomes, microvesicles, and apoptotic bodies, while plants produce tetraspanin-positive EVs, penetration 1-positive EVs, exocyst-positive organelle-derived EVs, and pollensomes [15,16].

**Figure 2:**
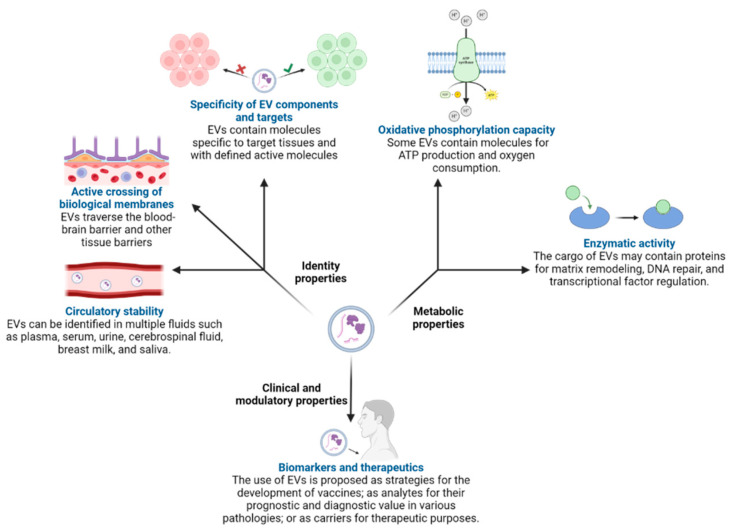
**Properties of bacterial EVs.** The properties of molecules exported in EVs can be grouped into three categories: identity properties, metabolic properties, and clinical/modulatory properties. Created with BioRender.com.

The observation of EVs by electron microscopy has demonstrated significant heterogeneity in their shape, being ovoid, spherical, semilunar, or truncated, and has allowed a deeper analysis of their biogenesis [11]. EVs are one of the main mechanisms of communication and modulation of the extracellular environment [11,17] and are regulators of pathogenesis during autologous and infectious processes [5,11]. Cells constantly produce EVs. However, their composition is altered in stress microenvironments, metabolic competition, phases of pathology, and detoxification. Vesiculation allows the export of biomolecules of different nature in the same compartment, which can interact with different cells even between unrelated organisms, so bacteria can use vesiculation as a synergistic or antagonistic communication system [12]. The molecules exported in EVs possess different properties [18], which can be grouped into three categories: identity properties, metabolic properties, and clinical/modulatory properties (Figure 2).

### 2.1. Extracellular Vesicles Produced by Human Cells

In humans, there are three main categories of EVs based on their origin, size, and composition:

1. **Exosomes** (20–200 nm) are of endosomal origin through the formation of multivesicular bodies in endosomes [11,12,17,19].

2. **Microvesicles** (150 nm–1 µm) are formed from the cell membrane [11,12,16,17,19].

3. **Apoptotic bodies** (1–5 µm) are released by dying cells and contain remnants of cellular organelles [10,19].

Classic exosomes express the tetraspanins CD9, CD63, and CD81, while non-classic exosomes express CD9 and CD63, but not CD81 [16,20,21,22]. Classic microvesicles express ADP-ribosylation factor 6 and CD35 and bind annexin A1 [16,21], while apopototic EVs bind annexin V [23,24]. However, there are still no universal markers for these EVs, and they are difficult to separate by experimental methods that rely on their physical properties [10]. Other EVs produced by human cells include autophagic EVs, stressed EVs, matrix vesicles, and exomeres [16]. In the context of infectious diseases, exosomes are mainly responsible for host–pathogen interactions [25]. 

### 2.2. Extracellular Vesicles Produced by Bacteria

Vesiculation in bacteria has been a widely studied process. Guerrero-Mandujano et al. propose vesiculation as a new type of secretion system that allows the release of lipids, proteins, peptides, nucleic acids, and biomolecules [12]. It was thought that bacteria of the genus *Mycobacterium* spp. and Gram-positive bacteria did not produce EVs due to the thickness of the mycobacterial and peptidoglycan cell walls, respectively. For these microorganisms, an enzyme-dependent vesiculation mechanism that degrades and reconstitutes the bacterial cell wall to allow the transit of EVs has been proposed. In addition, the presence of vesiculation pathways involving cell death have been recognized [26].

Bacterial EVs range from 20–200 nm and are classified by their origin [15] and release mechanism [27] into:

**Type B or non-lytic vesicles**. Includes outer membrane vesicles (OMVs), outer-inner membrane vesicles (OIMVs), and cytoplasmic membrane vesicles (CMVs).

**E-type or lytic vesicles**. Includes explosive cytoplasmic membrane vesicles (ECMV), explosive outer membrane vesicles (EOMV), and explosive outer-inner membrane vesicles (EOIMV) [15,27].

**Figure 3:**
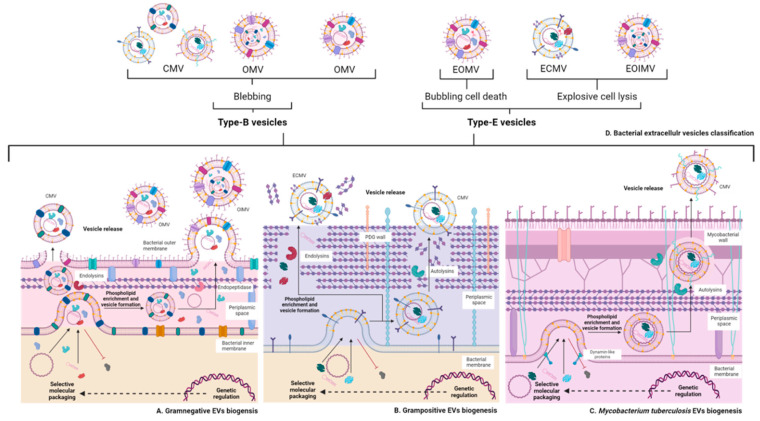
**Biogenesis and classification of bacterial EVs.** Vesiculation is a process regulated at the genetic level, involves the modification of membrane lipids and the activation of enzymes, and is stimulated by the microenvironment. (**A**) Gram-negative bacteria produce explosive cytoplasmic membrane vesicles (ECMV) or cytoplasmic membrane vesicles (CMV) from the inner membrane; these extracellular vesicles (EVs) contain mainly cytoplasmic material. Outer-inner membrane vesicle (OIMV) production involves the formation of EVs with cytoplasmic content from the inner membrane and periplasmic and outer membrane content. Outer membrane vesicles (OMVs) are the main form of vesiculation in Gram-negative bacteria; these EVs contain materials exported to the periplasmic space and wrapped in the outer membrane. (**B**) Gram-positive bacteria export their EVs through the peptidoglycan (PDG) cell wall by the action of autolysins (non-lytic pathway) or phage-encoded endolysins (lytic pathway). (**C**) *Mycobacterium tuberculosis* produces CMV by a mechanism very similar to that of other Gram-positive bacteria. In this group, the presence of dynamin-like proteins that facilitate vesicle formation has been identified. These EVs contain cytoplasm, membrane molecules, and mycobacterial cell wall lipoproteins. References: Brown et al. [28] (structure of bacterial membranes); Schwechheimer and Kuehn [29] and Charpentier et al. [30] (vesiculation in Gram-negative bacteria); Briaud and Carrol [31] (vesiculation in Gram-positive bacteria); Gupta et al. [32]; and Palacios et al. [14] (vesiculation in *M. tuberculosis*). Created with BioRender.com.

Toyofuku et al. collected evidence of the content of bacterial EVs. According to their classification, bacteria produce type B vesicles from the outer membrane, inner membrane, or both, without bacterial lysis. Bacteria also produce type E vesicles, which require bacterial lysis mediated by phage, enzymes, or during cell death for their release [27]. Figure 3 summarizes the biogenesis, classification, and composition of bacterial EVs. The type of bacterial EVs is dependent on the bacterial cell envelope architecture.

The production of lytic or non-lytic EVs responds to the adaptive mechanisms of the bacteria producing them. The Zavan group [33] analyzed the EVs of three *Pseudomonas aeruginosa* strains. They observed that EVs produced by lysis contain more DNA and proteins and a more diverse proteome. Interestingly, strains that produce EVs by lysis tend to be biofilm generators [34], and their EVs contain stress resistance molecules, antibiotics, and inflammatory mediators [35]. In turn, strains that export EVs without lysis contain virulence factors, such as porins OprB, OprD, and OprF, which are regulators of vesiculation in Gram-negative bacteria, in addition to bacteriocins [33,36].

Bacterial EVs are capable of mediating bacteria–bacteria and bacteria–host communication [12,37]. Bacterial EVs can generally contain proteins (enzymes, toxins, transporters, or metabolism proteins), nucleic acids, and virulence lipids [37]. Thanks to the MISEV guidelines [10], many bacterial EV populations have been studied with increasingly descriptive techniques, such as mass spectrometry and proteomic analysis [37]. In addition, technologies such as flow nanocytometry, nanoparticle tracking analysis, cryo-transmission electron microscopy, atomic force microscopy, and super-resolution microscopy have been used to analyze EV samples with single-vesicle resolution. These technologies allow a detailed characterization of EV subsets, which is useful in both functional studies and biomarker identification studies [38].

#### 2.2.1. Vesicles of Gram-Negative Bacteria

OMVs are the predominant EV population in Gram-negative bacteria [39]. However, there is recent evidence of OIMV production in *Shewanella vesicular* M7 [37], *P. aeruginosa* [33], *Klebsiella pneumoniae*, *Escherichia coli*, *Helicobacter pylori*, and *Acinetobacter baumannii* [40], suggesting that OIMV generation in Gram-negative bacteria is not an unusual event.

The envelope of Gram-negative bacteria consists of two membranes, an outer and an inner membrane, separated from each other by a thin peptidoglycan (PDG) layer in the periplasmic space [29]. The periplasmic space is where the OMV cargo is recruited and subsequently released from the membrane portion of the PDG layer and exported as a vesicle [29,39]. The representative component of the EVs of Gram-negative bacteria is lipopolysaccharide (LPS) [37] or endotoxin [41,42]. This molecule acts as a pyrogen and resistance factor to antimicrobial peptides and antibiotics [41,42]. At the immunological level, LPS is described as a potent activator of proinflammatory responses [42].

The formation of OIMVs requires the development of two cargo compartments. One develops from the bacterial cytoplasm into the vesicle portion formed by the inner membrane. The inner membrane vesicle subsequently migrates into the periplasmic space and the PDG is degraded by cleavage of the stability molecules between the outer membrane and the PDG layer, such as Braun’s lipoprotein [39]. The other cargo compartment forms when the outer membrane vesicle carries the inner membrane vesicle in its interior [29].

Characterization of the EVs proteome of *Shigella flexneri* 2A has identified at least 148 proteins and components in common with the EVs of Gram-negative bacteria [43]. OMV production has been widely described in Gram-negative bacteria, including the EVs of *Shigella dysenteriae* [44], *H. pylori* [45], *P. aeruginosa* [46,47], *K. pneumoniae* [40,48], *E. coli* [49,50] and other bacteria of this group [29,39]. The presence of molecules from the periplasmic space and the outer membrane has been demonstrated, which is a distinctive feature of OMVs.

The mechanisms of EV production have been studied in *Shigella* spp. [44], *P. aeruginosa* [46,48,51], and *K. pneumoniae* [40]. In two species of *Shigella* spp., different EV populations have been found, while in *S. dysenteriae*, OMV/CMV [44] have been isolated. In the EVs of *S. flexneri*, mainly molecules from the bacterial cytoplasm [43] have been characterized, so these EV could be OIMV or CMV. The mechanisms that promote vesiculation in *S. dysenteriae* are currently unknown. However, the process in this bacterium could be more conserved, as it has homology with vesiculation in other Gram-negative bacteria. Meanwhile, descriptions in *S. flexneri* suggest that the vesiculation process is controlled by a plasmid [52].

##### Effect of Growth Conditions on Vesiculation of Gram-Negative Bacteria

Culture media with stressful conditions, such as the presence of antibiotics, oxidizing molecules, hypoxia, or low nutritional intake, favor vesiculation. This response has been observed in most of the Gram-negative bacteria, such as *H. pylori* [53], *Salmonella* spp. [54], *Neisseria gonorrhoeae* [55], *A. baumannii* [56,57], or *P. aeruginosa* [58].

Iron is an indispensable microelement for bacterial growth. Some studies using minimal culture media devoid of this element increase vesiculation in *N. gonorrhoeae* [55], *A. baumannii* [56], or *E. coli* [50]. In the EVs of some of these bacteria, iron capture molecules such as enterobactin have been identified, indicating that one of the functions of vesiculation is the capture of nutrients [56].

The temperature of the host or growth medium influences vesiculation. An interesting example is *Serratia marcescens*, a bacterium isolated from some arthropods and whose pathology in humans is related to opportunistic urinary tract infections and sepsis. McMahon et al. reported that the highest vesiculation of *S. marcescens* occurs at temperatures below 30 °C, similar to growth conditions in arthropods, and the amount of EVs obtained at 37 °C is negligible. The authors evaluated the toxicity of EVs obtained below 30 °C on *Galleria mellonella* larvae, identifying an increase in TLR expression and larval mortality [59]. This experiment demonstrates that the species-specific pathogenicity of EVs depends on the factors that induce their production, such as temperature, so that microenvironment stress may also be suppressive to EV biogenesis.

The host microenvironment influences vesiculation processes. For example, *Campylobacter jejuni* does not possess the same secretion systems described in other enteric bacteria, so EV production has been proposed as one of the main mechanisms of pathogenicity [60]. The production of *C. jejuni* EVs enriched in virulence factors such as lipids and membrane proteins is favored in the presence of a bile salt, sodium taurocholate [61]. EVs produced in the presence of sodium taurocholate favor the adhesion of *C. jejuni* to the intestinal epithelium [62]. Similarly, *Salmonella enterica* subsp. *enterica* serovar Typhimurium cultures that simulate the conditions of intracellular growth and the intestinal invasion phase favor the production of EVs with islands of pathogenicity [54,63].

Therefore, vesiculation of Gram-negative bacteria is a highly regulated process that requires modification of membrane lipids and LPS components. It is influenced by changes in temperature and favored by the environments to which the bacteria have adapted, such as the host organism. The selective pressure of the host is a mediating factor for the content of the EVs.

#### 2.2.2. Vesicles of Gram-Positive Bacteria

Vesiculation in Gram-positive bacteria had been little described, because such a process would require cell death [13,37,64]. However, studies in *Bacillus cereus*, *Bacillus subtilis*, *Bacillus anthracis*, *Staphylococcus aureus*, *Listeria monocytogenes*, *Streptococcus pneumoniae*, and *Clostridium perfringens* demonstrated vesiculation in Gram-positive bacteria [13,64]. For Gram-positive bacteria to export their EVs requires multiple steps, from the selection of cytoplasmic components and the formation of a vesicle from the cell membrane to its transport through the cell wall [13].

It was not until 2022 that Jeong et al. observed vesiculation in Gram-positive bacteria using super-resolution stochastic optical reconstruction microscopy (STORM). Jeong et al. identified EVs with membrane and bacterial cell wall components and vesicles formed only by the cell membrane, suggesting that this bacterial group presents more than one vesiculation mechanism [13,27,65].

The molecules responsible for mediating the transit of EVs in Gram-positive bacteria are diverse. Enzymes that degrade the PDG cell wall are called autolysins or endolysins. Examples are the phenol-soluble modulins and PDG hydrolase from *S. aureus*, the endolysin encoded in a *B. subtilis* prophage, or the phage-derived endolysins from *S. pneumoniae* [13].

The generic molecules identified in the EVs of Gram-positive bacteria are PDG and components of the bacterial cytoplasm [37]. Teichoic acids, lipoteichoic acids, and PDG stand out among the cell wall components. PDG is, in weight-to-weight ratio with respect to LPS, a molecule with less inflammatory activity [64]. When analyzing PDG digestion derivatives, it has been observed that some PDG chains associated with teichoic and lipoteichoic acids initiate inflammatory responses [64], suggesting that components of the bacterial cytoplasm or molecules associated with PDG may be the main mediators of pathogenicity. This idea is supported by studies that have identified (in EVs) cytotoxic molecules from *B. anthracis* [13], the presence of α-hemolysin from *S. aureus*, hyaluronate lyase from *Propionibacterium acnes*, or listeriolysin O from *L. monocytogenes* [66].

In Gram-positive bacteria, it is common to find the generation of EVs mediated by autolysins that degrade and modulate vesicle transit or by phage-encoded endolysins that also degrade the bacterial cell wall, but with lysis of the bacteria. In the latter example, bacterial lysis implies that the vesicle cargo is more random [13,28,31].

## 3. Role of Extracellular Vesicles in Host Interactions with Gram-Negative and Gram-Positive Bacteria

In this work, we selected some Gram-negative and Gram-positive species that are representatives of WHO priority bacteria, according to Asokan et al. [67] and Bartlett et al. [68]. Bacterial infections are usually treated with antibiotics, so we first discuss the possible role of bacterial EVs in drug resistance.

### 3.1. Involvement of Bacterial Extracellular Vesicles in Drug Resistance

The mechanisms by which EVs of Gram-negative [69] and Gram-positive [70] bacteria participate in drug resistance involve the capture of molecules, a change in the composition of EVs, the transfer of resistance plasmids, or the presence of neutralizing enzymes. These effects are related to some studies showing that the presence of subinhibitory doses of antibiotics in the bacterial growth medium favors vesiculation in both Gram-negative bacteria, such as *A. baumannii* [71] and *K. pneumoniae* [72], and Gram-positive bacteria, such as *Enterococcus faecium* [73] and *S. aureus* [74,75]. In the following sections, we analyze individually the different resistance mechanisms mediated by EVs.

#### 3.1.1. Extracellular Vesicles Capture Drugs

Some models have proposed that the membrane of EVs could reduce the availability of antibiotic molecules in the medium. Marchant et al. used the *S. enterica* serovar Typhi strains ΔrfaE, ΔtlR, and ΔdegS, which are hyperproducers of EVs, to evaluate resistance to polymyxin B, an antibiotic that destabilizes the outer membrane of Gram-negative bacteria. The group found that EVs from ΔtlR and ΔdegS strains neutralized the antibiotic through their capture [69], an effect caused by the very presence of membrane lipids susceptible to binding by the drug. This effect has also been described in EVs from *A. baumanii* [76].

#### 3.1.2. Extracellular Vesicles Transfer Plasmids and Resistance Genes

EVs can transfer active resistance genes or molecules. Homologous transfer of antibiotic resistance plasmids by EVs has been described in *A. baumannii* [77] and *K. pneumoniae* [78,79,80] and between bacteria of different genera in *K. pneumoniae* [78,80] and *P. aeruginosa* [35]. The most studied EV-mediated transfer has been that of antibiotic resistance genes. In addition, we have described how EVs function as drug capture media targeting components of the outer membrane of Gram-negative bacteria. However, there is an intermediate mechanism that enhances the importance of the study of multidrug-resistant strains, and that is the transfer of genes for capsule synthesis observed in *K. pneumoniae* EVs [79].

#### 3.1.3. Extracellular Vesicles Contain Molecules That Confer Resistance to Antibiotics

EVs of drug-resistant strains of *N. gonorrhoeae* [81] and *A. baumannii* [71] contain β-lactamases that neutralize antibiotics such as penicillin, imipenem, and tetracycline, or antibiotic efflux pumps, as seen in extensively drug-resistant *K. pneumoniae* (XDR) [82,83,84], which promote resistance to eravacycline [85]. The presence of these resistance molecules in EVs generates a cross-protection effect in susceptible strains before they acquire a resistance plasmid [81], or as in the case of the efflux pump of *K. pneumoniae* XDR and its EVs, the expulsion of the drug would favor the growth of this strain over other susceptible strains by not degrading the antibiotic from the medium [82,83].

In *K. pneumoniae*, lipid change during vesiculation has been analyzed as part of the study of drug resistance mechanisms. In polymyxin-sensitive strains, the characterized EVs contain a higher concentration of glycerophospholipids, fatty acids, lysoglycerophosphate, and sphingolipids, which decrease bacterial membrane permeability. Lipidome analysis of EVs has been proposed as a screening tool to determine the sensitivity of some bacteria [72].

### 3.2. Extracellular Vesicles during Infection by Gram-Negative Bacteria

We can divide the effects of bacterial EVs into three stages: vesicle adhesion to host cells, vesicle internalization by target cells, and cellular response. The interaction of EVs with host cells can occur by binding to pattern recognition receptors (PRRs) or by the presence of adhesion molecules on the EVs. In some flagellated bacteria, flagellin facilitates adhesion; this has been demonstrated in *C. jejuni* EVs, which contain flagellin and, as hypothesized, facilitate adhesion to endothelial cells [86]. Other adhesion molecules identified in EVs are sialic acid adhesin (SabA), external inflammatory protein A (OipA), neutrophil-activating protein (HP-NAP), and adhesion lipoprotein (AlpA), which are found in *H. pylori* and mediate adhesion to the gastric epithelium [45].

The internalization or capture of *H. pylori* EVs depends on two main effects: the presence of VacA toxin [87] and the size of the EVs themselves. On the latter topic, Turnes et al. observed that smaller EVs (20–100 nm) enter cells via caveolin-mediated endocytosis, while larger EVs (90–450 nm) enter via micropinocytosis [88]. In *H. pylori* it has also been observed that epithelial cells internalize EVs using clathrin-dependent mechanisms [89]. In models using *E. coli*, it has been determined that the presence of LPS favors the internalization of EVs by lipid rafts. EVs could enter by membrane fusion; in models with *P. aeruginosa* and *S. aureus*, this entry pathway has been analyzed, which depends on the presence of cholesterol-rich regions [89].

Once EVs are internalized by host cells, many authors have observed that they have proinflammatory effects generally attributed to the presence of LPS. Internalization of EVs from NTHi [90] strains of *K. pneumoniae* [40] into respiratory epithelial cell lines or EVs from *H. pylori* into a gastric epithelial cell line [88,91] results in the production of IL-8, IL-6, and TNFα. The administration of EVs via NTHi aerosols in murine models of neutrophilic asthma [92] increases the release of IL-1β and IL-17. The inflammatory response promoted by EVs has also been evaluated in in vivo models of sepsis. Uropathogenic *E. coli* EVs alter cardiac muscle Ca^2+^ influx; EVs were detectable at the cardiac level after EV inoculation, generating tachycardia and thickening of the heart wall with increased TNFα and IL-6 [93].

Another component in the EVs of Gram-negative bacteria involved in the initiation of proinflammatory cytokine production is *H. pylori* vacuole-forming toxin (VacA) [45,87,94]. In addition to inflammation, *H. pylori* EVs have been observed to promote apoptosis of immune and epithelial cells.

A group of proteins extensively studied in EVs are porins, which are regulators of vesiculation and mediators of toxicity [95]. Porin B (PorB) in *N. gonorrhoeae* [96] EVs and the porin OmpA in *A. baumannii* and *Acinetobacter nosocomialis* EVs are involved in epithelial adhesion and colonization. Cells internalizing EVs loaded with these proteins show increased cell death in models of macrophage stimulation with *N. gonorrhoeae* [96] EVs or epithelial cells in models of sepsis caused by *A. baumanii* [97,98].

Vesiculation of *P. aeruginosa* is also regulated by porins. The study of the lytic and non-lytic vesiculation mechanism has provided insight into how these proteins are regulated. In *P. aeruginosa*, a quorum-sensing protein called *Pseudomonas* quinolone signal (PQS) favors non-lytic vesiculation [34,99,100]. This protein is regulated by the porins OprF and OprD [101], which are homologous to OmpA (identified in bacteria of the ESKAPE group, such as *A. baumannii* and *E. coli*) [46]. EVs generated by this non-lytic mechanism contain biofilm matrix-degrading enzymes [99]. Another *P. aeruginosa* vesiculation-associated porin, OprI, covalently binds the outer membrane to the PDG; the absence of this protein favors vesiculation due to the weakening of the binding bonds between the outer membrane and the PDG [46].

Bacterial EVs can interact with the central nervous system. *Haemophilus influenzae* type B EVs contain lipooligosaccharide (LOS), which promotes meningeal inflammation by increasing the permeability of the blood-brain barrier [102,103]. *H. pylori* EVs, whose ability to affect the permeability of the blood-brain barrier has not been demonstrated, are taken up by astrocytes. Astrocyte activation leads to the presence of amyloid precursors and to the development of pathology in Alzheimer’s disease models [104,105].

*H. pylori* EVs have been mainly studied for their relationship to gastric cancer. Increased cell proliferation has been described after stimulation with *H. pylori* EVs. This proliferation is caused by *H. pylori*’s main virulence factors: cytotoxin A (CagA), VacA, and urease [91,94]. CagA is a bacterial oncoprotein, VacA is responsible for mitochondrial failure and cell death, and urease allows bacterial survival in acidic media [106].

In *S.* Typhimurium, PhoP-PhoQ proteins participate in LPS remodeling and increase vesiculation. PhoPQ recruits factor H of the complement system, which inhibits C3b. The PhoPQ system is expressed during intracellular growth of *S.* Typhimurium so that these EVs can facilitate bacterial survival after host cell death [107]. Porins and LPS from Gram-negative bacteria are strong promoters of inflammation. However, not all EVs from Gram-negative bacteria are proinflammatory, since the EVs of *Bacteroides thetaiotaomicron* and *B. fragilis* promote anti-inflammatory responses in colitis models [29].

### 3.3. Extracellular Vesicles during Infection by Gram-Positive Bacteria

The PDG coat of Gram-positive bacteria is poorly immunogenic. The ability of these bacteria to be recognized by host cells is dependent on molecules such as teichoic acids [64]. In this bacterial group, CMV production by lysis or cell wall degradation is the main form of vesiculation [27]. Olaya et al. identified that *S. pneumoniae* can release EVs with components of the bacterial cytoplasm, such as the transcriptional regulator MaIX and the virulence-associated surface protein PspA, without requiring bacterial lysis [108]. On the other hand, lytic vesiculation could be phage-mediated, since phage and autolysin (AtlA) genes have been identified in *E. faecium* EVs [109] that could be involved in promoting EV generation [110].

Some molecules identified in the EVs of *S. aureus* are vesiculation regulators and damage mediators, such as phenol-soluble α- and β-modulins and hemolysins, which cause lysis of erythrocytes, neutrophils, monocytes, and epithelial cells [111]. At the same time, phenol-soluble α-modulin is responsible for the export of EVs [27], indicating that the pathological role of some molecules is secondary to their function as mediators of bacterial communication.

Lipidation of bacterial cell wall lipoproteins appears to be a regulator of vesiculation. Kopparapu et al. found that wild-type strains of *S. aureus* produced a greater number of EVs, with an average diameter of 65 nm, whereas mutated strains that do not lipidate or do not contain surface proteins produced numerous populations of EVs with an average diameter of ~140 nm [112]. Temperature also affects the vesiculation of Gram-positive bacteria. For example, *S. aureus* produces homogeneous populations of EVs at 37–40 °C [111], which contain more virulence factors than EVs obtained below 30 °C [113], but at this temperature, there is hyper vesiculation [111] and a more significant variability in the sizes of EVs [113]. In this bacterial group, growth in media devoid of iron or supplemented with growth inhibitors such as antibiotics or oxidizing molecules also increases vesiculation [74,111].

Proteomic analysis of *S. aureus* EVs by Uppu et al. found 54 proteins that could not be detected in culture supernatant [114]. Some important proteins found in *S. aureus* EVs are α-hemolysin, leukocidin, superantigens, and phenol-soluble modulins and enzymes possibly involved in vesiculation [111,115]. Some proteins identified in *S. aureus* EVs do not possess signal peptides, indicating that they are complete proteins with biological activity [111]. The molecules characterized in *S. aureus* EVs have been grouped into four families: pore-forming toxins, superantigens, exfoliative toxins, proteases [111], and RNAs [116] such as SsrA and RsaC, which are related to antibiotic resistance, or RNAIII toxin [117]. In *S. pneumoniae* EVs, endonucleases that degrade extracellular traps such as TatD have been identified [118,119]. These allow greater invasiveness in murine sepsis models.

Biofilm generation is a survival strategy that allows bacterial clustering and protection against antimicrobials. As part of microbial competition, some bacteria produce bacteriocins or growth inhibitors. Im et al. [36] have shown that the treatment of a polystyrene surface with *S. aureus* EVs decreases its hydrophobicity and inhibits biofilm generation by some ESKAPE group bacteria (*E. faecium*, *K. pneumoniae*, and *A. baumannii*) [36], while *S. aureus* biofilm formation is unaffected.

One of the most outstanding properties of *S. aureus* EVs is the function of their components, among which the functional β-lactamase BlaZ has been characterized but its gene has not. In contrast to the antibiotic sequestering activity observed in *S.* Typhimurium EVs [69], *S. aureus* EVs were not able to capture antibiotics, so the resistance associated with EVs relies on the presence of neutralizing enzymes [120]. Another effect associated with EVs was the induction of apoptosis in HEp-2 cells, which was not described when treating the same cell line with lysed EVs, indicating that the whole vesicle is necessary for its internalization by susceptible cells [121].

**Figure 4:**
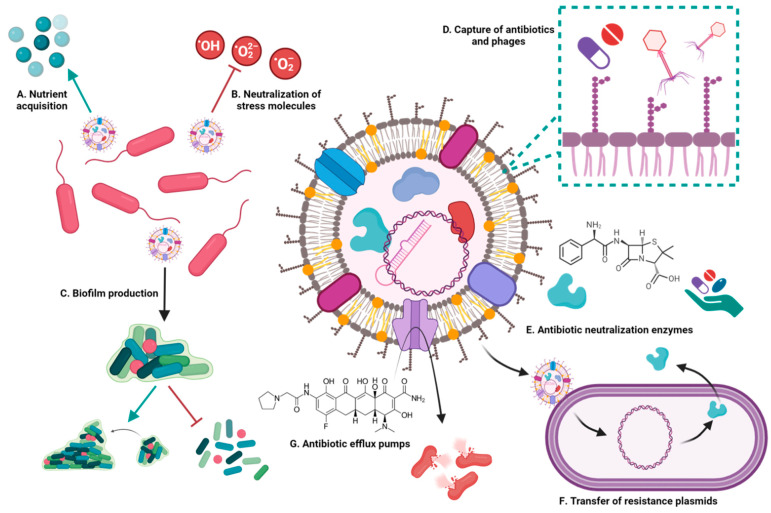
**Protective mechanisms associated with bacterial vesicles.** EVs contain protection and survival molecules. (**A**) Some molecules facilitate nutrient capture. (**B**) The content of EVs can neutralize free radicals. (**C**) EV proteins can induce biofilm formation or decrease the hydrophobicity from surfaces to inhibit biofilm formation. (**D**) A defense mechanism against drugs and phages is their capture by the presence of target molecules or viral receptors on EVs. (**E**,**F**) The transfer of resistance plasmids or the presence of resistance proteins allows survival against antibiotics. (**G**) The presence of antibiotic efflux pumps could be mediators of bacterial resistance and increased antibiotic bioavailability for susceptible bacteria. Created with BioRender.com.

The main roles of EVs from Gram-negative and Gram-positive bacteria in protection and survival are summarized in Figure 4.

## 4. Extracellular Vesicles in Other Medically Important Bacteria

### 4.1. Extracellular Vesicles in Sporulating Bacteria: Bacillus and Clostridium

Within the phylum Bacillota, accepted as a synonym of Firmicutes, we find two classes: Bacilli, which is represented by strict aerobic or facultative anaerobic bacteria, and Clostridia, which includes strictly anaerobic bacteria [122]. In these classes, we find spore-producing genera, of which those of clinical importance are *Bacillus* spp. and *Clostridium* spp. The genus *Bacillus* has as its main representatives *B. anthracis*, *B. cereus*, *B. subtilis*, and *B. licheniformis*, while the genus *Clostridium* includes *C. botulinum*, *C. tetani*, *C. perfringens*, and *C. difficile*, among other species [123].

Vesiculation in Firmicutes was first described by Dorward in 1990. Early morphological characterization assays of EVs in this phylum by Brown in 2015 indicate that many Firmicutes export EVs ranging in size from 20–150 nm. However, some clinically important species, such as *Bacillus* spp. and *C. perfringens*, give rise to large EVs, up to 400 nm [11,124].

The presence of prophage genes coding for endolysins in *Bacillus* spp. suggests that this genus performs lytic vesiculation [11,27]. Within the molecular cargo of *Bacillus* spp. and *Clostridium* spp. EVs, nucleic acids, PDG-degrading enzymes, antibiotic resistance proteins, toxins, virulence factors, immunogenic compounds [11], and the lethal factor of *B. anthracis* [125], have been described.

Existing evidence does not point to a link between spore generation and vesiculation. However, since EVs participate in nutrient capture, an increase in sporulation signals could repress vesiculation by reducing bacterial metabolism and the need for nutrient capture, while an increase in vesiculation signals could inhibit sporulation by promoting bacterial growth.

Brown et al. reported vesiculation in *B. subtilis* and *B. anthracis*, where they characterized a dual-function component of EV metabolism. Their work discovered that cultures of *B. subtilis* strains producing the antimicrobial peptide surfactin resulted in lower quantification of EVs in the medium compared with non-surfactin-producing strains. However, this was not due to the number of EVs released but because surfactin is able to degrade the membrane of EVs from both *B. subtilis* and other bacteria [124]. Therefore, the group believes that these cell membrane-targeted peptides would function as antimicrobial agents, mediators of the release of EV contents, or both.

Imaging studies have demonstrated the transit of *B. subtilis* EVs through intestinal epithelial cells [126]. Buchacher et al. indicate that EV capture depends on the presence of cholesterol microdomains in the host epithelium [127]. However, it has been shown that, like other Gram-positive bacteria, *B. cereus* EVs transport active molecules such as sphingomyelinase, phospholipase C, and enterotoxin Nhe into the intestinal epithelium. These molecules were not significantly identified in the culture supernatant, highlighting EVs as vehicles of molecule transport [127].

Vesiculation of *C. difficile* was analyzed by Nichlas et al., who noted that TcdA and TcdB toxins were not present in EVs of laboratory strains or clinical isolates. However, exposure of intestinal cells to *C. difficile* EVs induces a dose-dependent proinflammatory and cytotoxic response [125]. Caballano et al. found that despite the absence of TcdA and TcdB in *C. difficile* EVs, their epithelial toxicity involves the expression of apoptosis-related genes (CASP3), an effect that was not distinguished in non-toxigenic strains of *C. difficile* [128].

In summary, the EVs obtained in the growth phases of sporulating bacteria are not different from the EVs of other non-sporulating Gram-positive bacteria, except for their size. It is necessary to describe the effect of stressful conditions on vesiculation in this bacterial group, as well as the relationship between spore formation and EV synthesis.

### 4.2. Extracellular Vesicles of Gram-Negative Bacteria Devoid of LPS: Treponema Pallidum

Although some species of the genus *Treponema* are classified as Gram-negative due to the presence of an inner and outer membrane separated by a PDG layer, some authors consider that this classification criterion is not sufficient [129], because the lack of LPS on the outer membrane raises questions about the participation of membrane molecules in the pathogenesis associated with this bacterial genus.

Many studies that analyze the EVs of *T. pallidum* date to between 1994 and 2005. Therefore, their results are difficult to compare because they did not follow the current guidelines for the analysis of EVs. The obtention and, in general, the study of *T. pallidum* EVs is highly complicated because this species is not cultivable. Blanco et al. implemented a method for obtaining EVs based on ultracentrifugation and sucrose gradients of a *T. pallidum* suspension isolated from infected rabbit testes [130]. The *T. pallidum* EVs studied contain very low-density proteins, which were classified by weight into 17, 28, 31, 45, and 65 kDa. The 17 and 45 kDa proteins are highly immunogenic and the 65 and 31 kDa proteins correspond to the *Treponema* rare outer membrane proteins (TROMPs) [130,131], which are a group of membrane proteins of Gram-negative bacteria that in *T. pallidum* are up to 100 times less expressed [131]. This low level of expression could constitute a mechanism to evade recognition by antibodies [130]. This same research group purified an anti-EV antibody from *T. pallidum*, which had a protective effect when administered as a therapy before the challenge with the bacterium [132].

Vesiculation in *T. pallidum* raises questions about the relationship between LPS and the vesiculation capacity of Gram-negative bacteria. In the phylogenetic analysis of some species of *Treponema* spp. it is revealed that *T. pallidum* is contemporary with *T. vincentii* [133], so as some authors cite, vesiculation is a conserved and primitive process, and it is valid to question the functions of LPS on bacterial metabolism and communication.

### 4.3. Extracellular Vesicles of Mycobacterium Tuberculosis

Tuberculosis, a disease caused by *Mycobacterium tuberculosis*, is considered the most lethal disease associated with a single microorganism, making it one of the major health emergencies worldwide [134,135]. The growing evidence of vesiculation in Gram-negative bacteria facilitated interest in investigating vesiculation in Gram-positive bacteria [13,65,66]. It prompted the investigation of vesiculation mechanisms in *M. tuberculosis*, especially after Marsollier et al. isolated EVs of *Mycobacterium ulcerans* from skin biopsies of infected mice [14]. The central conundrum about *M. tuberculosis* vesiculation was that this bacterium has a complex cell wall that would not allow the transit of EVs or would permit their expulsion only by bacterial lysis [136].

Prados-Rosales et al. were pioneers in evidencing vesiculation in *M. tuberculosis*. They demonstrated that the administration of *M. tuberculosis*-derived EVs intratracheally to mice induced exacerbation of the infectious process and lung damage [137], while subcutaneous administration of these EVs reduced *M. tuberculosis* load, highlighting their potential as vaccines [138]. Since then, numerous studies have been carried out on the effects of *M. tuberculosis* EVs on the host. However, few studies have analyzed the process of their production [32].

Following the work of Prados-Rosales on *M. tuberculosis* vesiculation in iron-depleted media [137], Rath et al. identified that the vesiculogenesis and immune response regulator (*VirR*) gene is a repressor of vesiculation, and that iron deficiency—a condition that favors vesiculation—decreases *VirR* expression [139]. White et al. reported that the activation of the SenX3-RegX3 system by the inhibition of the phosphate transporter gene *PstA1* is an activator of vesiculation in *M. tuberculosis* [140]. The mechanism by which these genes inhibit or activate vesiculation is unknown.

In 2023, Gupta et al. reported that a set of dynamin-like proteins, IniA and IniC (encoded in the *iniBAC* operon), initiate the formation of EVs at the cell membrane level [32]. IniA/C deficiency in *M. tuberculosis* decreases its vesiculation, and iron deficiency activates the iniBAC operon [14,32]. Finally, the strongest hypothesis on the export of EVs in *M. tuberculosis* considers that, as with Gram-positive bacteria, *M. tuberculosis* produces EVs by transit of the vesicle through the bacterial cell wall, mediated by autolysins [14,115]. The idea of the transit of EVs in *M. tuberculosis* by the action of autolysins was proposed after the characterization of the lipidome of the EVs showed an enrichment of membrane lipoproteins and very few components of the mycobacterial cell wall [137].

The case of *M. tuberculosis* EVs is very interesting, as numerous immunogenic molecules and modulators of the immune response have been identified in these vesicles. The main virulence factors of *M. tuberculosis* are its lipids. Phosphatidylinositol, phosphatidylinositol mannosides, phosphatidylethanolamine, cardiolipins or liporabinomannan (LAM) [137] have been found in EVs. Within the proteomic analysis of EVs, the presence of the lipoproteins LpqH and LprG [137,141,142] stands out.

*M. tuberculosis* EVs contain multiple lipoproteins that are PRR ligands, especially for TLR-2, such as LpqH, LprG, LprA, PhoS1, and LAM [137], which could lead to the maturation of dendritic cells (DCs). Our group identified this effect by stimulating DCs with EVs derived from *M. tuberculosis*-infected neutrophils. DCs can activate CD4+ T cells [143] and increase proinflammatory molecules such as IL-1β, IL-6, IL-12, TNFα and CXCL1, and the anti-inflammatory cytokine IL-10 [14,137]. However, groups such as Athman et al. have reported that EVs from macrophages infected with *M. tuberculosis* contain agonists for TLR-2 capable of inducing a Th1 response. Among these agonists is LAM [144], which can be transported to CD4+ T cells and block their activation [145].

Research on *M. tuberculosis* vesicles could lead to numerous applications. Vesicles obtained in the laboratory could function as a means of immune activation/inhibition and be exploited in the development of vaccines. At the same time, EVs produced during tuberculosis would allow us to analyze their participation in the pathogenesis of the disease or use them in the search for diagnostic markers [146].

## 5. Immunological Effects of Bacterial Extracellular Vesicles

Bacteria-derived EVs contain common components that define the vesicles of Gram-positive and Gram-negative bacteria and mycobacteria. Many immune responses induced by EVs are similar among the three bacterial groups, suggesting that host cell recognition molecules can bind different pathogen-associated molecular patterns (PAMPs) in EVs, but the activation pathways are convergent. EVs contain PAMPs such as DNA, RNA, LPS, PDG, membrane proteins, lipoproteins, or virulence factors.

Recognition of EVs via TLR appears to be a conserved response, even in invertebrate hosts. As we saw, *S. marcescens* EVs increase the expression of TLRs in their invertebrate host, the larval *G. mellonella* [59]. Enterotoxigenic *E. coli* (ETEC) EVs from clinical isolates (and to a lesser extent those of commensal *E. coli*) [147] and those from *P. aeruginosa* [89] are mainly recognized by the TLR-4 pathway. The lipoproteins of *S. aureus* EVs are recognized by TLR-2 and, to a lesser extent, by TLR-4 (binding PDG derivatives) and TLR-9 (recognizing unmethylated CpG motifs) [75]. In *S. aureus*, membrane lipids are the ligand of TLR-4, as strains deficient in lipidation activity of lipoproteins (Δlgt) induce the production of IL-6, MIP-2 and TNFα in macrophages by a TLR-2 dependent mechanism [112,148]. As for *M. tuberculosis*, the LpqH, LprG, PhoS1, and LAM lipoproteins are ligands of TLR-2 [137,144].

Laakmann et al. analyzed the effects of *K. pneumoniae, E. coli*, *S.* Typhimurium, and *S. pneumoniae* EVs on endothelial cells, which express ribonuclease 1 (RNase1) to regulate the inflammatory response. Their results show that the EVs from the Gram-negative microorganisms decreased endothelial expression of RNase1 in a TLR4-dependent manner, which could result in an exacerbation of the inflammatory response in sepsis [149].

In models with Gram-negative bacteria, stimulation with bacterial EVs induces proinflammatory responses. Stimulation of a gastric epithelial cell line with *H. pylori* EVs leads to their activation via MyD88, with subsequent induction of NF-κB, ending in the secretion of TNFα, IFNγ, IL-5, IL-6, IL-12, and IL-8 [45,150,151] or the anti-inflammatory cytokine IL-10 [152]. EVs from *E. coli* [49,93], *H. influenzae* Type B, and NTHi [102,103] also induce NF-κB expression and an increase in proinflammatory cytokine production. In intestinal epithelial cells, *C. jejuni* EVs induce the production of IL-8, IL6, hBD-3, and TNFα [60,153]. Intestinal epithelial cell lines treated with *E. faecium* EVs suffer from cytotoxicity and production of IL1β, IL-6, IL-8, and MCP-1 [73]. *P. aeruginosa* EV-stimulated human macrophages produce IL-1β, IL-8, and IL-6, and upregulation of the IL-10 gene has also been described [154]. *K pneumoniae* EV-stimulated lung epithelium also increases the production of IL-8, IL-6, IL-1β, and TNFα [48].

Vesiculation in Gram-negative bacteria of the same species allows us to analyze that virulence factors in EVs of pathogenic strains can influence the activation of inflammation. Behrouzi et al. reported that EVs of ETEC induce a greater inflammatory response than a commensal strain of *E. coli*. The inflammatory response is activated by a mechanism that is also dependent on TLR-4 [147].

Some virulence factors that have been analyzed in detail are flagellar proteins and porins. The flagellin present in the EVs of *S.* Typhimurium, *E. coli*, and *P. aeruginosa* is recognized by the macrophage receptor NLRC4, which produces IL-1β more efficiently than bacterial varieties devoid of flagellin [155]. It is possible that the patterns of some proteins recognized in the cytoplasm induce similar responses. In the section in which we described the vesiculation mechanisms, we mentioned that porins are proteins that regulate vesicle synthesis in Gram-negative bacteria in addition to being highly immunogenic. For example, the OmpA porin of *N. gonorrhoeae* and *A. baumannii*, present in EVs, prompts the secretion of IL-1α/IL-1β155, IL-6, MIP-1α, and neutrophil infiltration in mice and cell cultures treated with EVs [77,156]. The EVs of *A. baumannii* devoid of the OmpA gene induce a lower production of IL-6, NLRP3, and IL-1β in mice [76]. EVs from Gram-negative bacteria, such as *H. pylori*, induce the production of ROS [45]. However, catalase activity, which neutralizes species such as H_2_O_2_ and NaClO, has been identified in this same bacterium [157].

As mentioned in the initial sections of this work, the PDG layer of Gram-positive bacteria has poor immunogenicity [64], so other molecules have been proposed as immunological mediators of the EVs of this bacterial group. Studies in *S. aureus* have shown that cell wall-associated lipoproteins can induce inflammatory responses [112]. The internalization of *S. pneumoniae* and *S. aureus* EVs by pulmonary epithelial cells leads to the activation of keratinocytes, macrophages, and DCs via NF-κB [158,159,160]. *S. pneumoniae* EVs are internalized by macrophages and induce the activation of NF-κB [159]. *S. pneumoniae* EVs increase the production of TNFα by dendritic cells [158] and, after being systemically inoculated in mice, they increased the plasma levels of CCL5, CCL2 and CXCL10 [159]. *B. cereus* EVs induce TNFα production in monocytes and cause enterocyte lysis [127]. Exposure of intestinal and hepatic cells to *C. difficile* EVs induces the production of IL-1β, IL-6, IL-8, and MCP-1 [125,128].

Allen et al. obtained EVs from neutrophils treated with live and heat-inactivated S. *aureus*, wherein the EVs from the live bacterium-treated neutrophils activated the production of IL-6 and IL-1β in macrophages [161]. In models of atopic dermatitis, stimulation with *S. aureus* EVs promoted an increase in IL-6, IL-4, IL-5, IFNγ, IL-17, and IgG with infiltration of mast cells and eosinophils [162]. The DNA and RNA identified in *S. aureus* EVs are recognized by PRRs such as TLR-7, TLR-8, and TLR-9, leading to the degradation and antigenic processing of EVs secreted in endosomal spaces [163].

Infected cells express cytokines that mostly lead to the initiation of the inflammatory response. In some cases, such as EVs produced by *M. tuberculosis*, immune response-promoting or evasive responses may be observed. The treatment of mice with *M. tuberculosis* EVs-containing aerosols inhibits T cell activation through a mechanism involving the blocking of IL-2 production by vesicle LAM [145]. In addition, RNA contained in *M. tuberculosis* EVs has also been shown to activate RIG-1 receptors toward IRF3 expression, which promotes phagosome maturation, enhancing macrophage bactericidal activity [89]. These examples help us transition from the inflammatory response mediated by EVs to the presentation of antigens to initiate the adaptive response [25,164].

Since the 1990s, some studies have identified molecules for antigen presentation in B-cell-derived EVs, which can activate T cells [163]. EVs increase antigen presentation by DCs in tuberculosis models, and this increased antigen presentation is attributed to the high density of antigens in the EVs [14,143]. In addition, DC-derived EVs increase the interaction surface available to T cells [163]. The activation and maturation of DCs leads to increased expressions of MHC-II, CD80, and CD86, and mature DCs are producers of TNFα and IL-12 [165,166,167]. Antigen-presenting EVs may be exosomes formed as multivesicular bodies of infected cells during phagocytosis of pathogenic bacteria or after activation of intracellular PRRs following the internalization of pathogen-derived EVs. EVs expressed by antigen-presenting cells such as DCs contain, in addition to the molecules of the pathogen, presentation and co-stimulation molecules such as MHC-I, MHC-II, or CD86 that can initiate the adaptive response [164].

An immunization of mice with NTHi EVs resulted in an increase in the level of IgG1 and IgG2 antibodies, in addition to elevated levels of IL-10, IL-4, and IFNγ. The level of IL-4 stood out from the rest, indicating that the adaptive response induced by EVs is mostly humoral [168]. Studies on *Shigella* spp. have led to the postulation of some proteinases that are potentially protective [169,170]. Oral [171,172] or oral/ocular/nasal mucous membranes immunization with *S. flexneri* EVs [173] have generated protective responses to a challenge with lethal doses of the virulent bacterium [170].

An immunization of mice with *H. pylori* EVs induced an increase in the level of IgG1 and IgG2 and protection of the animals from microbial challenge [174]. The presence of IgG1 and IgG2 classes is attributed to a change in the profile of a Th1 response (with IgG2 expression) toward a Th2 response (assessed by IgG1 level) [174]. Induction of a Th1 and Th17 response has also been reported after EV immunization of ETEC and intestinal isolates of commensal *E. coli* [175,176,177] and *K. pneumoniae* in a murine model of sepsis. The researchers observed a decrease in bacterial load after the microbial challenge and an increase in IgG titer. Macrophages stimulated with EVs produced IL-6 and TNFα, while T cells increased their IFNγ production [178].

Antibodies generated following EV immunization of Gram-negative bacteria can confer maternal-neonatal protection. Mitra et al. performed immunization modeling with EVs of *Shigella boydii* type 4 [172] and with a multi-serotype formulation of EVs of *S. dysenteriae* 1, *S. flexneri* 2a, 3a and 6, and *Shigella sonnei* [171]. Oral immunization of female mice induced the production of anti-EV IgG1, IgG2a, and IgG2b173. Subsequently, the experimental animals underwent the gestation period, and the passive immune transfer to progeny was evaluated. All the progeny animals survived the challenge with the virulent strain of *S. boydii* type 4 [172]. Both breast milk and neonatal animals had opsonizing antibodies, and mice were protected against the challenge of the wild strains *S. dysenteriae* 1, *S. flexneri* 2a, 3a, and 6, and *S. sonnei* [171,172].

Proteins such as PsaA, PBP5, LysM, DdcP, PpiC, and the SagA protein, which has been studied individually for vaccine development [110], have been characterized in *E. faecium* EVs. After the immunization of rabbits with EVs, Wagner’s group analyzed, in a passive transfer model, the use of the antibodies obtained, observing the protection of the inoculated animals after three doses [179]. Meanwhile, while analyzing the EVs of Gram-positive bacteria, Olaya et al. immunized mice with *S. pneumoniae* EVs, which were subsequently challenged with the wild-type bacteria. The presence of IgG antibodies and a high rate of protection and survival were observed in the immunized mice [108].

The immunogenicity of EVs produced by macrophages infected by *S.* Typhimurium [180] and by strains of this bacterium deficient in flagellin [181] has been evaluated. An IgG and IgA antibody-dependent response in mucous membranes has been observed [180,181] and was detectable for up to 12 weeks in a scheme of three immunizations [180]. In addition, EVs from strains devoid of flagellin induced a cross-protective response against *S. enterica* subsp. *enterica* serovars Choleraesuis and Enteritidis for at least five weeks [181].

Antigens from purified *S. aureus* EVs have been reported to induce Th1-like protective responses in murine models [182], but EVs from drug-resistant strains may contain fewer immunogenic molecules or a higher content of evasion molecules. Asano et al. did not observe protection of mice immunized with methicillin-resistant *S. aureus* EVs. Instead, the death of the experimental animals was documented [75].

**Figure 5:**
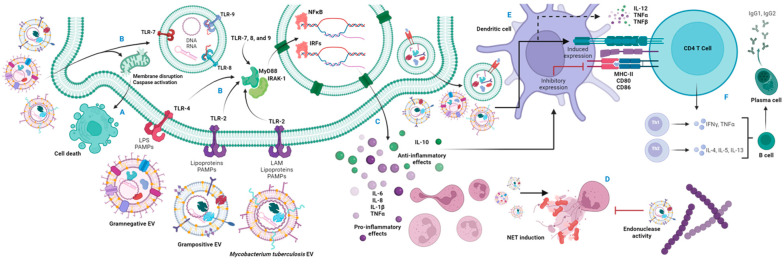
**Immunological effects of bacterial extracellular vesicles.** EVs can enter cells by protein-dependent or membrane fusion mechanisms and trigger responses in the cells that capture them. (**A**) Some EV molecules are virulence factors that activate cell death. (**B**) EVs contain PAMPs that can be recognized by cell membrane TLRs and TLRs in cytoplasmic compartments. (**C**) TLR signaling leads to the production of proinflammatory cytokines (TNFα, IL-1β, IL-6, IL-8). (**D**) These cytokines lead to the recruitment of neutrophils, which can produce NETs by the activity of EVs; however, some species, such as *S. pneumoniae*, express EVs with endonucleases to survive extracellular traps. (**E**,**F**) Anti-inflammatory cytokines (such as IL-10) produced by cells activated by EVs can inhibit the expression of co-stimulatory molecules; however, it has also been reported that bacterial EVs, or EVs derived from infected cells, stimulate the maturation of DCs, which present bacterial antigens to CD4^+^ T cells, which can induce cellular responses or lead to activation and antibody production by B cells. References: Schwechheimer and Kuehn [29]; Briaud and Carrol [31]; Gan et al. [89]; Prados-Rosales et al. [137]; Palacios et al. [14]; and Vázquez-Flores et al. [143]. Created with BioRender.com.

In this section, we summarized various characteristics of the host–pathogen relationship linked to EVs, from the mechanisms that induce their production and the protection they provide to the bacteria that produce them, to the generation of responses by the host cells (Figure 5 and Table 1), which can promote protection against infection or even cause damage to the host. In the next section, we consider EVs as promising platforms for developing new diagnostic, therapeutic, and preventive strategies.

## 6. Therapeutic Proposals Based on Extracellular Vesicles

### 6.1. Treatments Based on the Use of Extracellular Vesicles

Some working groups have explored EVs of microorganisms as drug carriers. Many of the proposed treatments seek to evade cell membrane selectivity through the ability of EVs to be internalized by host or bacterial cells. Kadurugamuwa et al. used *S. flexneri* EVs as gentamicin transporters to treat an infection of an enterocyte cell line. They demonstrated that gentamicin-containing EVs were more efficient than soluble gentamicin in decreasing the *S. flexneri* bacterial load of infected cells [183]. The high penetration of *S. flexneri* EVs into the enterocyte cell line was associated with their content of IpaB, IpaC, and IpaD, three proteins that participate in cell invasion [183].

Other groups have taken up the principle of antibiotic carriers designed by Kadurugamuwa et al. [183]. Gao et al. designed vancomycin- or rifampicin-loaded nanoparticles embedded in *S. aureus* EVs, which were captured by infected macrophages in an in vitro model and improved the prognosis of infected mice in an in vivo model [184].

The phenomenon of toxicity induced by EVs has been used to evaluate their effectiveness against cancer. *C. jejuni* vesicles expressing cytolethal distending toxin (CDT) inhibit the proliferation of the colorectal adenocarcinoma cell line Caco-2 by inducing apoptosis and autophagy [185]. A recent paper suggests that unmodified *E. coli* EVs have an anti-tumor effect. Jin et al. purified EVs from an *E. coli* strain and observed that EVs suppressed proliferation and had a cytotoxic effect on a neuroblastoma cell line and in a murine model of neuroblastoma [186]. Mice treated with four intravenous EV inoculations showed reduced cancer-associated symptoms and decreased tumor growth and angiogenesis [186]. This group mentions that it tracked some EV populations in other organs, such as the liver, but does not report toxicity trials for this [186].

The studies presented in this section suggest several ways in which EVs could be used as treatments for infections and cancers, but more work in this area is necessary to determine whether these findings have clinical relevance.

### 6.2. Vaccines Based on Extracellular Vesicles or Their Components

The development of vaccines based on EVs presents several challenges, with reproducibility in obtaining EVs and homogeneous characterization being one of the most complicated to address. Banadkoki et al. developed a conjugate vaccine of purified EVs of *P. aeruginosa* and diphtheria toxin, which was evaluated in a mouse burn infection model. Their model obtained a decrease in bacterial load, inflammatory response, and infiltration [187]. Li et al. produced EVs from a *P. aeruginosa* strain that lacked multiple virulence factors. They demonstrated that these EVs had lower cytotoxicity and toxicity in mice than the EVs from a wild-type *P. aeruginosa* strain, but retained their immunogenicity [188].

Li’s group developed a vaccine proposal based on EVs obtained from a *P. aeruginosa* strain devoid of toxicity-associated virulence factor genes. These EVs were enriched with the proteins PcrV (a component of the type-III secretion system) and HitAT (related to iron capture) and administered to mice. The response induced by these vesicles generated cross-protection against various variants of *P. aeruginosa*, characterized by a Th1/Th17 profile [188].

*S.* Typhimurium EVs have been proposed as a vaccine against *S. flexneri*. By fusing *S.* Typhimurium EVs with *S. flexneri* polysaccharide determinants, protection of mice against a challenge with virulent bacteria was obtained [189]. Other authors have proposed using EVs from *Burkholderia pseudomallei* as an adjuvant for a heat-killed *S.* Typhimurium-based vaccine, and this formulation protected mice against a lethal dose of the bacterium [190].

Another proposal for vaccines against Gram-negative bacteria was presented by Badmasti et al., who developed EVs by bioengineering using two proteins from *A. baumannii*: OmpA, responsible for regulating vesiculation [97], adhesion, invasion, and cell lysis [191]; and Bap, associated with biofilm production and adhesion. In their model, the immunization of mice led to elevated IgG titers, an increase in the level of IL-4 and IFNγ, and a decreased bacterial load in a sepsis model [191]. Recently, Matthias et al. synthesized *Neisseria meningitidis* EVs that did not contain the virulence factors PorA, PorB, and RmpM and observed that, in a murine model, these vesicles protected against challenges with *N. gonorrhoeae* in a three immunization scheme [192].

Li et al. introduced a novel method for generating bacterial biomimetic vesicles (BBVs) from *K. pneumoniae*. In this method, high pressure is used to force the bacteria to pass through small slots. As a result, the bacterial membranes break and release the cytoplasmic content, and then re-assemble into vesicles that contain very little DNA or cytoplasmic proteins [193]. This BBV generation method could be an alternative for vesicle-based vaccine development, since immunization with *K. pneumoniae* BBVs induced DC maturation, increased levels of IL-6 and TNFα, IgG production, decreased neutrophil infiltration and increased survival in a murine model of sepsis [193].

The development of vaccines against Gram-positive bacteria based on EVs stands out for one aspect: the most efficient antigen-carrying platform is the EVs of Gram-negative bacteria. A proposed method using *E. coli* vesicles loaded with *S. pneumoniae* OmpA or CLM37 has conferred protection against pneumococcal infection [194]. Muralinath et al. employed *S.* Typhimurium EVs loaded with *S. pneumoniae* PspA protein. The purified PspA protein did not confer protection when used to immunize mice [195], indicating that the characteristics of *S.* Typhimurium EVs make them adjuvants that enhance the response against *S. pneumoniae* antigens.

Knowing the antigenic variation between strains of the same species, Irene et al. proposed the design of polyvalent vaccines against *S. aureus*. Their group designed a vaccine with five *S. aureus* antigens presented in *E. coli* EVs, which generated protection against infection in lungs, kidneys, skin, and models of murine sepsis [160,196]. König et al. proposed a vaccine with four virulence proteins from *S. aureus*, which generated an opsonizing antibody response in mice and protection against skin challenges and in models of renal abscesses and sepsis [197].

Previous work has led to very promising models, such as that developed by Sun et al., who designed a vaccine based on bioengineered *E. coli* EVs using the Plug-and-Display system targeting three *S. aureus* antigens (EsxA, Sbi, and SpA), which induced a more efficient Th1 response than the use of peptides with an aluminum salt adjuvant [198]. On the other hand, Zanella et al. created the strain *E. coli* BL21(DE3)Δ60, which is a hyperproducer of EVs devoid of endogenous proteins, which allows the binding of up to 5–30% of proteins of interest [199]. The group linked *S. aureus* FhuD2 and HlaH35L proteins to EVs (the immunogenicity of these proteins was described by Irene et al. [196]) and immunized mice, which showed an increase in the level of IgGs. The antibodies were neutralizing for *S. aureus* HlaH35L in a hemolysis model [199]. The appropriate selection of antigens for vaccine development continues to be a critical step for the development of multivalent vaccines based on EVs. However, Zanella’s work [199] poses an attractive model for the generation of EVs that can be obtained with homogeneous characteristics, is a reproducible method, and allows the design of multivalent vaccines, such as the one proposed by Irene et al. [196].

There are currently four licensed EV-based vaccines against *N. meningitidis* [200] and at least five EV-based vaccines in clinical trials [https://clinicaltrials.gov] (Table 2). Three of these vaccines use EVs produced by genetically modified bacteria that are hyperproducers of vesicles derived from their outer membranes. These EVs are known as generalized modules for membrane antigens (GMMA) [201].

**Figure 6:**
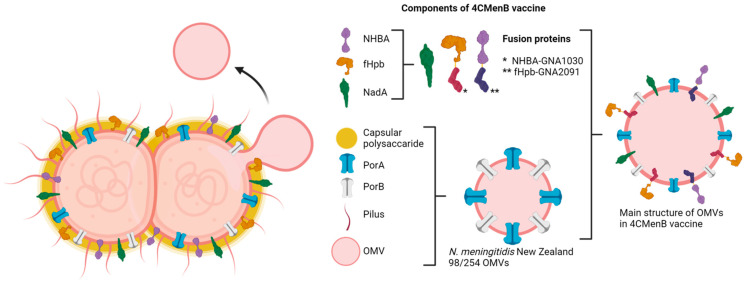
**4CMenB vaccine formulation.** The vaccine contains five proteins: NadA and the NHBA-GNA1030 and fHpb-GNA2091 fusion proteins. These proteins are inserted into the EVs of *N. meningitidis* New Zealand 98/254, which contain the PorA and PorB porins. Created with BioRender.com.

GSK’s group B 4CMenB *N. meningitidis* vaccine *(four-component recombinant protein-based serogroup B meningococcus*) was licensed for use in Europe in 2013 [202], and was first applied in the UK in 2015. As of 2019, no significant adverse effects had been detected [203]. To develop this vaccine, the genome of *N. meningitidis* was characterized, identifying its proteome. Through bioinformatic assays, its surface proteins were identified, which were transformed into *E. coli* to evaluate its potential to induce antibodies. Twenty-eight candidate proteins were selected, and the vaccine was formulated with the proteins that showed the best responses. The final design of the 4CMenB vaccine contains the protein NadA and the NHBA-GNA1030 and fHpb-GNA2091 fusion proteins. These proteins were inserted into the EVs of *N. meningitidis* New Zealand 98/254, which contain the PorA and PorB porins [204,205,206] (Figure 6). The use of EVs in this vaccine substantially improved its immunogenicity and provided coverage against more strains [205].

The 4CMenB vaccine offers 66–91% protection. It can be used in children from 2 months of age and has recently been approved for use in individuals aged 10–25 years [203]. Although the vaccine has been reported to confer protection against different strains of *N. meningitidis*, there are also strains associated with invasive processes against which the 4CMenB vaccine does not confer protection [203]. The MeNZB vaccine, based on EV of *N. meningitidis* group B, offers cross-protection against *N. gonorrhoeae*, and this protection against several species has also been observed with 4CMenB [203,207]. In conclusion, the use of bioengineered vesicles for vaccine development is a promising strategy with proven effects in the vaccines licensed for use, and bacterial EVs have been shown to be efficient adjuvants that can be modified to present antigens of pathogens of interest.

As mentioned above, the main challenges in the development of vaccines based on EVs are the difficulties in obtaining homogeneous EVs in a consistent manner. However, these challenges have been successfully overcome in other areas of lipid-based nanotechnology. Lipid nanoparticles (LNPs) are transport and delivery platforms for drugs, proteins, and nucleic acids. LNPs range from 40 to 1000 nm in size, they are stable, and they are easily produced at large scale at low cost [208]. LNPs are made of defined proportions of purified solid lipids with melting points higher than 37 °C [209], or liquid lipids [208]. LNPs can also contain ionizable cationic lipids (to transport nucleic acids) [210] or polyethylene glycol (to reduce their recognition by phagocytic cells and their clearance by renal filtration) [211]. LNPs can enter the host cells through macropinocytosis, clathrin, or caveolae endocytosis [212]. LNPs have become a reliable vehicle for nucleic acids. They are approved by the FDA for use in Onpattro, which is an interference RNA encapsulated in LPNs that is used for the treatment of polyneuropathy [213]. They are also approved for their use in two mRNA-LNP vaccines against COVID-19, the BNT162b2 vaccine from Pfizer-BioNTech [214], and the mRNA-1273 vaccine from Moderna [215], both of which are currently in clinical use. The success of LNPs suggests that fully synthetic vesicles could be used as carriers of EV antigens and adjuvants and to develop more robust vaccines.

## 7. Conclusions and Prospects

Vesiculation is an ancient form of cellular communication that is found in the three domains of life. In pathogenic bacteria, EVs represent an integral component of pathogenicity mechanisms, and many EV components have several effects on the host immune system. In this review, we summarized what is currently known about these effects: Bacterial EVs can contain virulence factors that induce host cell death and PAMPs that activate innate immune receptors and lead to the production of proinflammatory cytokines (such as TNFα, IL-1β, and IL6) and inflammation. However, anti-inflammatory cytokines (such as IL-10) can also be produced in response to bacterial EVs. Bacterial EVs stimulate the maturation of DCs, which present bacterial antigens to CD4+ T cells, leading to adaptive immune responses and antibody production by B cells. In addition to the PAMPs that induce DC maturation, bacterial EVs also carry bacterial antigens, which further promote the activation of CD4+ T cells.

All of these characteristics make bacterial EVs an attractive platform for vaccine development. However, some challenges must be overcome during the development of bacterial EV-based vaccines. To reduce the potential toxicity of bacterial EVs, the EVs can be produced from bacterial strains that lack virulence factors. To increase EV immunogenicity, the EVs can be produced from bioengineered bacterial strains that express high levels of known antigens, and this strategy could be used to produce multivalent vaccines. To increase the reproducibility of EV composition, artificial vesicles can be produced, including BBVs and LNPs. With these strategies, and with a deeper understanding of the effects of bacterial EVs on the immune system, bacterial EVs could be used to develop more effective vaccines against clinically relevant infections.

## Figures and Tables

**Table 1:** Immunological effects of bacterial extracellular vesicles.

Bacteria Producing the EVs	Target Cell	Effects on the Immune System	References
**EVs contain PAMPs recognized by PRRs**			
*S. aureus*	Mouse macrophages	Lipoproteins recognized by TLR2 and TLR4 induce IL-6, MIP-2, and TNFα production	[113,149]
	HEK-Blue reporter cells	RNA and DNA detected by TLR7, TLR8 and TLR9	[164]
*M. tuberculosis*	Mouse macrophages	LpqH, LprG, PhoS1 and LAM lipoproteins recognized by TLR2	[138,145]
	Mouse macrophages	RNA activates RIG-1 receptors, inducing IRF3 expression, and an increase in macrophage bactericidal activity	[90]
**EVs have a proinflammatory effect**			
*H. pylori*	Human gastric epithelial cells	Induction of NF-κB results in the production of TNFα, IFNγ, IL-5, IL-6, IL-12 and IL-8	[51,151,152]
*C. jejuni*	Human intestinal epithelial cells	Secretion of TNFα, IL-8, IL-6 and hBD-3	[66,154]
*P. aeruginosa*	Human macrophages	Production of IL-1β, IL-6 and IL-8	[155]
*K. pneumoniae*	Human bronchial epithelial cells	Increased TNFα, IL-8, IL-6 and IL1β production	[54]
*C. difficile*	Human intestinal and hepatic cells	Induces the production of MCP-1, IL-1β, IL-6 and IL-8	[126,129]
**EVs have an anti-inflammatory effect**			
*H. pylori*	Human monocytes	Secretion of IL-10 induced by MyD88-dependent signaling pathway	[153]
*P. aeruginosa*	Human lung macrophages	Upregulation of IL-10 expression	[155]
*S. pneumoniae*	Murine dendritic cells	Increased IL-10 production	[159]
**EVs contain virulence factors**			
*S.* Typhimurium	Mouse macrophages	Flagellin increases IL-1β production, via NLRC4	[156]
*A. baumannii*	Murine model	OmpA porin stimulates IL-1α/IL-1β, IL-6, MIP-1α, and neutrophil infiltration	[78,157]
**EVs induce adaptative immune responses**			
Non-typeable H. *influenzae*	Murine model	Increased levels of IgG1, IgG2, IL-10, IL-4 and IFNγ	[169]
*H. pylori*	Murine model	Increased levels of IgG1 and IgG2, related to a change toward a Th2 profile	[175]
*E. coli*	Murine model	Induction of Th1 and Th17 responses	[176,177,178]
*S. pneumoniae*	Murine model	Induction of IgG production, protection and survival	[109]
*K. pneumoniae*	Murine model	Increased IgG production and decrease of bacterial load	[179]
	Mouse macrophages	Increased IFNγ production by T cells	[179]
EV from *S.* Typhimurium-infected macrophages	Murine model	Induction of IgA and IgG in mucous membranes	[181,182]
EV from *M. tuberculosis*-infected neutrophils	Human dendritic cells	Induction of DC maturation and increased IFNγ production by T cells	[144]

**Table 2:** Licensed EV-based vaccines, and EV-based vaccines in clinical trials.

Vaccine	Components	Targeted Disease	Phase
VA-MENGO-BC	EVs derived from *N. meningitidis* group B and capsular polysaccharide from *N. meningitidis* group C	Meningococcal infections	Licensed
MenBvac	EVs derived from *N. meningitidis* P1.7,16 strains	Meningococcal infections	Licensed
MeNZB	EVs derived from *N. meningitidis* New Zealand 98/254 strain	Meningococcal infections	Licensed
GSK 4CMenB	EVs derived from *N. meningitidis* New Zealand 98/254 strain with additional antigens	Meningococcal infections	Licensed
GSK meningococcal group B vaccine administered concomitantly with GSK meningococcal MenACWY conjugate vaccine	Recombinant membrane proteins (rMenB) with EVs from the New Zealand B strain, administered concomitantly with a quadrivalent meningococcal tetanus toxoid conjugate vaccine (MenACWY)	Meningococcal infections	3
GSK meningococcal group B vaccine and 13-valent pneumococcal vaccine administered concomitantly with routine infant vaccines	Recombinant membrane proteins (rMenB) with EVs from the New Zealand B strain, administered concomitantly with pneumococcal conjugate vaccine (PCV 13) and other routine infant vaccines	Meningococcal and other infections	3
GSK Vaccines Institute for Global Health (GVGH) invasive non-typhoidal salmonellosis (iNTS)-GMMA	EVs derived from modified *S.* Typhimurium and *S.* Enteritidis	Invasive non-typhoidal salmonellosis	2
GVGH iNTS-GMMA-typhoid Vi polysaccharide-conjugate vaccines (TCV)	EVs derived from modified *S.* Typhimurium and *S.* Enteritidis, with the addition of TCV	Invasive non-typhoidal salmonellosis and typhoid fever	1/2
GSK *N. gonorrhoeae* GMMA (NgG)	*N. gonorrhoeae* GMMA	Gonorrhea	1/2

## Data Availability

Not applicable.
